# Distinctive adsorption and transport behaviors of short-chain versus long-chain perfluoroalkyl acids in a river sediment

**DOI:** 10.1007/s11356-024-35725-1

**Published:** 2024-12-07

**Authors:** Na Liu, Mengyan Li

**Affiliations:** 1 Department of Chemistry and Environmental Science, New Jersey Institute of Technology, Newark, NJ 07102 USA; 2 National Engineering Research Center for Efficient Utilization of Soil and Fertilizer Resources, College of Resources and Environment, Shandong Agricultural University, Tai’An, 271018 PR China

**Keywords:** Sediment, Point source, PFOS, PFBA, Hotspot

## Abstract

Perfluoroalkyl acids (PFAAs) embrace perfluorooctanesulfonic acid (PFOS), perfluorooctanoic acid (PFOA), and other concerning chemicals of different chain length and terminal moieties. PFAAs can leach from municipal wastewater facilities as point sources discharging into rivers and receiving streams. In this study, we investigated the adsorption and transport behaviors of six select PFAAs in a Hudson River (USA) sediment in both batch and mesocosm studies. The adsorption capacities single and dual solute systems followed the order: PFBA < PFHxA ≈ PFBS < PFHxS < PFOA << PFOS. Mesocosm experiment that receives a continuous point source discharge of a mixture of these six PFAAs reached equilibrium after 4 weeks of operation. Total adsorbed PFAAs in the sediment was extracted and analyzed, following PFHxS (0.85 mg, 20.4%) ≈ PFBS (0.92 mg, 21.7%) < PFOA (1.02 mg, 27.3%) ≈ PFHxA (1.04 mg, 29.8%) < PFBA (1.12 mg, 30.1%) << PFOS (1.55 mg, 39.2%). PFOS showed highest adsorption, concentrating on the surface layer. Noticeably, two short-chain PFAAs, PFBA and PFHxA, were found with high vertical mobility, partitioning into deeper sediment. Two hotspots for PFAA sediment contamination were formed near the sediment surface downstream from the point source, providing new prospects to guide PFAA sediment cleanup and monitoring.

**Supplementary Information:**

The online version contains supplementary material available at 10.1007/s11356-024-35725-1.

**Figure:**
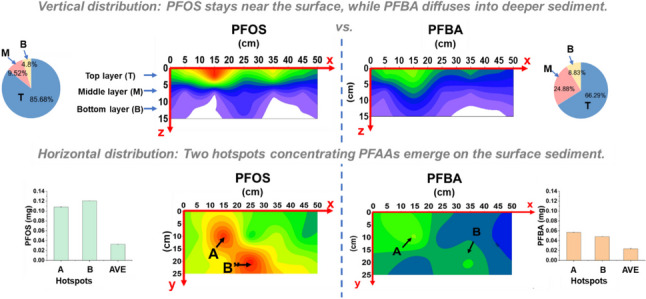
Graphical abstract

## Introduction

Perfluoroalkyl acids (PFAAs), the most persistent per- and polyfluoroalkyl substances (PFAS), consist of two major groups, perfluoroalkyl carboxylic acids (PFCAs) and perfluoroalkane sulfonates (PFSAs) (Hall et al. 2020). To date, many point sources have been recognized for their continuous discharge of PFAAs and associated compounds, including wastewater treatment plants (WWTPs), manufacturing factories, and firefighter training sites where aqueous film-forming foams (AFFFs) have been extensively used (Ahrens et al. 2011, Ahrens et al. 2015, Gonzalez et al. 2021, Lindstrom et al. 2011, Vries et al. 2017, Wu et al. 2022). Because of their persistent and hydrophilic characteristics, PFAAs can transport far away from the source to remote areas through water and air currents (Ahrens et al. 2009, Ahrens et al. 2015, Becker et al. 2008, Davis et al. 2007, Paul et al. 2009, Prevedouros et al. 2006, Shoeib et al. 2006, Taniyasu et al. 2013). As a consequence, PFAAs have been found widely distributed in the environment, wildlife, and humans (Ahrens and Bundschuh 2015, Eak et al. 2020, Giesy and Kannan 2001).

According to different carbon lengths, PFAAs are categorized into long-chain (PFCAs with eight or more carbons, and PFSAs with six or more carbons) and short-chain (PFCAs with seven or fewer carbons and PFSAs with five or fewer carbons) PFAAs. Most studies and regulations focus on long-chain PFAAs, particularly perfluorooctanesulfonate (PFOS) and perfluorooctanoic acid (PFOA), considering their carcinogenicity and potential to accumulate in fatty tissues and other body compartments. In 2017, the United States Environmental Protection Agency (U.S. EPA) released a stringent Drinking Water Health Advisories for PFOA and PFOS (EPA 2017). Recently, the U.S. EPA has legislated the regulation of these two PFAAs with maximum contaminant levels as low as 4 ng/L (USEPA 2024). Manufacture of PFOA, PFOS, and their precursors has been enlisted in Stockholm Convention, and banned in North American and European countries in the past decade (Li et al. 2020, Vierke et al. 2012, Wilhelm et al. 2010). These regulations resulted in a worldwide PFAS manufacturing shift from long-chain to short-chain PFAAs and associated compounds. Furthermore, increasing studies have revealed the occurrence of short-chain PFAAs from the partial breakdown of long-chain PFAAs and precursor compounds (Gewurtz et al. 2019, Liu and Avendaño 2013, Mumtaz et al. 2019). With improved coverage of analytical approaches, detection of short-chain PFAAs has been reported at impacted sites and diverse environmental compartments of growing frequencies (Andersson et al. 2019, Brendel et al. 2018, Dauchy et al. 2019, Guelfo and Higgins 2013, Hall et al. 2020, Higgins et al. 2017, Lang et al. 2017, Li et al. 2020, Lin et al. 2020, Liu et al. 2019, Wen et al. 2020, Wu et al. 2022). Therefore, increasing attention has been drawn to short-chain PFAAs since their environmental behaviors and health impacts have been scarcely studied (Gewurtz et al. 2019, Mumtaz et al. 2019).

Riverine, estuary, and other surface aquatic environments are perceived as major sinks of PFAAs that are consistently discharged from underground contaminated sites, industrial portals, and municipal WWTPs located along the water streams (Lindstrom et al. 2011, Mak et al. 2009, Nakata et al. 2006). Sorption to the benthic sediment is an important process that affect PFAA behaviors in surface water environments (Ahrens et al. 2010, Higgins and Luthy 2006, Zhao et al. 2014, Zhao et al. 2020). Recent batch studies revealed the significant adsorption of PFAAs to sediments and other aquatic materials, reducing the downstream transport of PFAAs (Dauchy et al. 2018, Dauchy 2019, Dauchy et al. 2019). Despite these batch tests, investigation of PFAAs transport behavior is still in a relatively nascent stage. The transport behaviors of PFAAs in the sediment can be intricate along all dimensions (i.e., longitudinal, vertical, and horizontal), warranting a systematic investigation of environment-relevant conditions where PFAAs of varying chain lengths and functional moieties commingle.

**Fig. 1:**
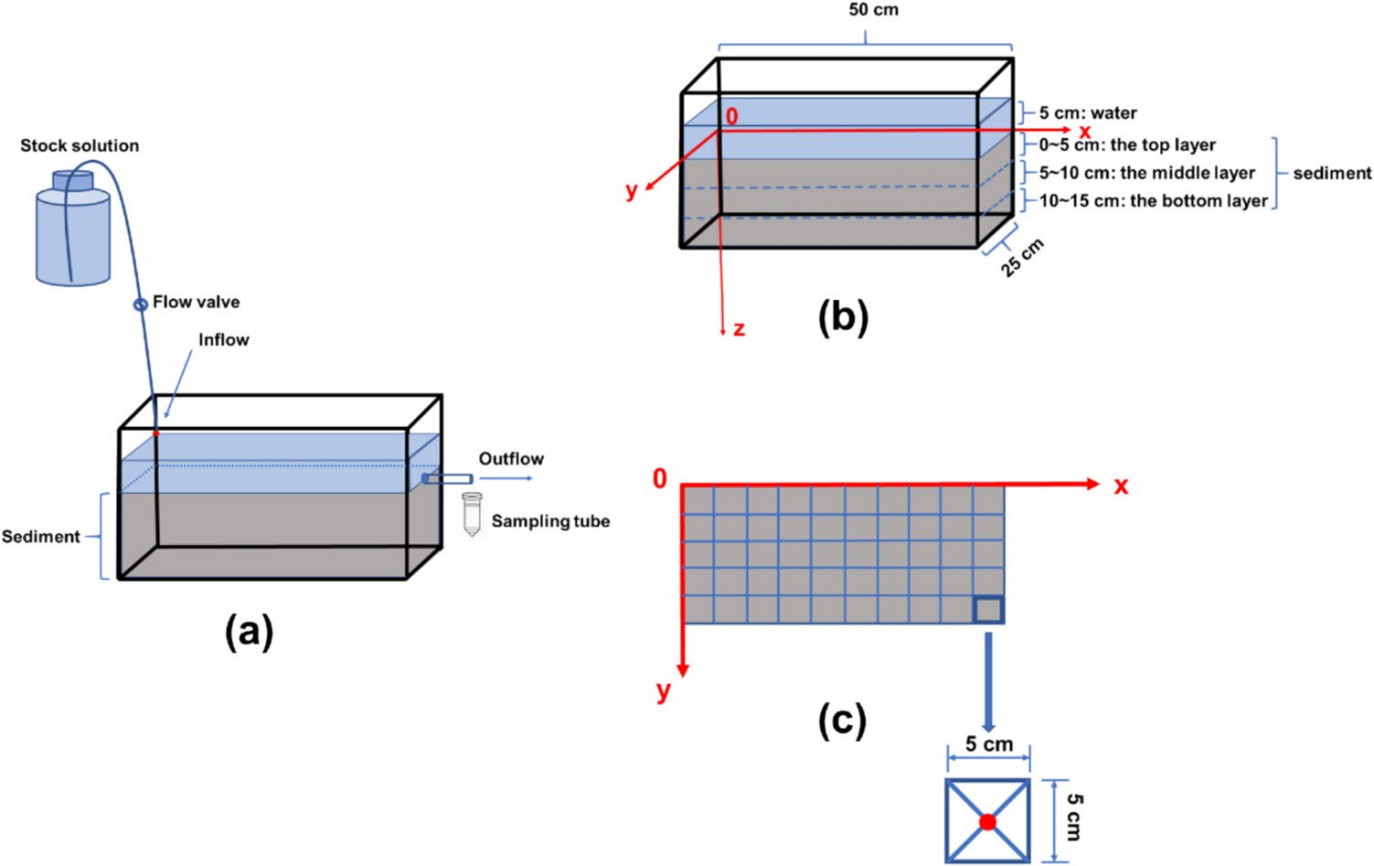
Experimental apparatus for the mesocosm system (**a**) and the establishment of 3-D coordinates in the sediment (**b**). The red dot indicates the point source where the mixture of six PFAAs is continuously discharged. After the adsorption reached equilibrium, the sediment was sectioned into 5 × 5 × 5 cm cubes (**c**). Sediment samples at the center point of each cube were analyzed for PFAA contamination. From the surface to the bottom, the sediment is evenly segregated into three layers: top, middle, and bottom. Each layer is 5 cm in depth

Sorption to soil or sediment is considered the primary retention mechanism which may influence PFAA distribution and transport in the field (Borthakur et al. 2021, Luthy et al. 1997). In this study, we set up a mesocosm (Fig. 1) to evaluate the 3-D distribution and transport of six different PFAAs compounds in the sediment-water system with a continuous point source. C4, C6, and C8 PFCAs and PFSAs were selected to represent PFAAs of different chain lengths and functional groups. Sediment was collected from the Hudson River watershed as its water quality and natural biota are endangered by the prevailing contamination of PFAAs based on recent national and regional surveys and reports (Brase et al. 2022). As a unique tidal estuary ecosystem, the Hudson River also serves a dense population and diverse aquacultures within the NY-NJ-CT tristate area in the USA, posing intensive exposure risks to local residents and natural biota. By comparing the PFAA profiles in corresponding sediment and water samples, the transport behaviors of six selected PFAAs were investigated using both batch and mesocosm assays. The results of this study can provide a guideline for the sediment remediation in surface water environments that receive a continuous point source of mixed PFAA contamination.

## Methods and materials

### Chemicals and materials

Six PFAAs were purchased from Sigma-Aldrich (St. Louis, MO), including three short-chain compounds: perfluorobutanoic acid (PFBA, C_4_F_7_O_2_H), perfluorobutanesulfonic acid (PFBS, C_4_F_9_SO_3_H), perfluorohexane acid (PFHxA, C_6_F_11_O_2_H), and three long-chain compounds: perfluorohexane sulfonate (PFHxS, C_6_F_13_SO_3_H), perfluorooctanoate acid (PFOA, C_8_F_15_O_2_H), and perfluorooctane sulfonate (PFOS, C_8_F_17_SO_3_H). The physicochemical properties of these six PFAAs are outlined in Table S1. The mixed PFAA standard (≥98% purity) and mass labeled PFHxS (M-PFHxS) standard were purchased from Wellington Laboratories (Ontario, Canada). Calibration curves were prepared with the mixed PFAA standard to achieve a series of concentrations ranging from 0.1 to 100 µg/L using Milli-Q water. The stock PFAA solution for the transport and competitive adsorption (100 µg/L) were prepared by mixing the appropriate quantity of PFAAs (PFBA, PFBS, PFHxA, PFHxS, PFOA, PFOS) with Milli-Q water prior to immediate use.

### Sediment sampling and pretreatment

Sediment samples from the Hudson River, USA, were used as the porous media. The sampling site was located next to the Ross Dock Picnic Area in New Jersey (40.86° N, 73.96° E). River depths were highly variable, with navigable channel depths averaging 12 m and a maximum depth of 61 m. Sediment samples were collected at the time of low tide. The surface sediment (0–20 cm) was grabbed by a Bottom Sampler acc. to Lenz (Hydro-Bios, Germany). Sediments were drained and homogenized using a clean concrete mixer for further use. A portion of homogenized sediment were stored at −20 ℃ for 24 h and then freeze-dried. The weight difference before and after the freeze-drying procedure was used to estimate the sediment moisture content.

### Batch experiments

Adsorption of PFAAs by the sediment was assessed using a batch equilibrium method at a sediment-water ratio of 333 g/L. Briefly, 30 mL Milli-Q water spiked with individual PFAA at the initial concentration of 100 µg/L and 10 g freeze-dried sediment samples were added into 50-mL polypropylene (PP) centrifuge tubes. After vortexed for 30 s, all tubes were shaken at 180 rpm for 7 days (168 h) to reach adsorption equilibrium at the room temperature. The sediment-based partition coefficient (*K*_d_) (L kg^−1^) of each PFAA was calculated following Eq. (1).

1 $${K}_{d}=\frac{{C}_{s}}{{C}_{w}}$$

where *C*_s_ (ng/kg dry weight (dw)) is the PFAA concentration in the sediment, and *C*_w_ is the dissolved PFAA concentration in water (ng/L).

Similarly, dual solute batch experiment was carried out to investigate the competitive adsorption between PFHxS and the other five PFAAs of interest. In the dual solute system, the initial concentration of each PFAA was 100 µg/L and all tubes were shaken at 180 rpm for 7 days (168 h). Ratio (α) of the equilibrium sorption amount (*q*′_e_) in the PFHxS-PFAA dual solute system and that (q_e_) *in single solute system was calculated following Eq. *(2)*.*

2 $$\alpha =\frac{{q{\prime}}_{e}}{{q}_{e}}$$

where *q*′_e_ (ng/g dry weight (dw)) is the equilibrium sorption amount in the PFHxS-PFAA dual solute system, and *q*_e_ is the equilibrium sorption amount in the single solute system.

### Mesocosm studies

A mesocosm (Fig. 1) was set up in a glass tank (50 cm length × 30 cm height × 25 cm width) to mimic the aquatic system that receives a consistent inflow of a mixture of six PFAAs. The tank system was packed under the water-saturated condition with the homogenized sediments collected from the Hudson River. The depth of the sediment was approximately 15 cm packed at the bottom of the tank to mimic the river benthos (Fig. 1). Then, Milli-Q water was filled until 5 cm above the surface of the sediment. At one corner of the water tank, the inflow solution that consisted of 100 µg/L mixed PFAAs (PFBA, PFBS, PFHxA, PFHxS, PFOA, PFOS) was introduced to the water at the continuous flow rate of 1 mL/min (Fig. 1a). The outflow was collected daily from the other longitudinal end of the tank for the analysis of PFAAs. After 4 weeks of operation, the concentration of the outflow became stable and rose to the level as in the inflow (Fig. S1), and thus the operation of this mesocosm was terminated by the cessation of the inflow. After the water was completely drained, the sediment was separated into cubic units that were 5 cm length ×5 cm height × 5 cm width as depicted in Fig. 1c. For each cubic unit, the center sediment sample (~3 g) was collected in the diagonal intersection of each cubic unit using sample spoon in triplicate. Sediment samples were then transferred to 50-mL polypropylene tubes and stored at −20 °C for > 12 h before they were freeze-dried and grounded into fine powders (<0.15 mm) for PFAA extraction.

### PFAA extraction and analysis

PFAAs in the sediments were extracted using a previously described method (Higgins et al. 2005) with minor modifications. Briefly, 2 g of freeze-dried sediment samples were transferred to 50-mL polypropylene tubes. 10 µL M-PFHxS stock solution (500 ng/mL) was spiked to the sediment samples as the mass-labeled surrogate for the extraction recovery analysis before extraction (equivalent to 2.5 ng/g). Then, 10 mL of 0.1% acetic acid solution (methanol: 1% acetic acid, 90:10 (v/v)) was added. Each tube was vortexed for 15 min and then sonicated at a frequency of 100 Hz for 20 min in a water bath heated at 60 °C. After sonication, samples were centrifuged at 5,000 rpm for 15 min and the supernatant was transferred into a new 50-mL PP tube. The sediment residuals were extracted two more times with 5 mL of 0.1% acetic acid solution. For each sample, all three solvent extracts (approximately 20 mL) were combined, concentrated under constant pure nitrogen flow to almost dryness, and dissolved with 1 mL methanol. After filtered with a disposable 0.22-μm PES filter, sediment extracts were then analyzed using LC/MS/MS as described below. Water samples for the PFAA analysis were measured directly after filtered using a disposable 0.22-μm PES filter.

PFAA concentrations were analyzed using a 1290 Infinity II HPLC system in tandem with 6470A triple quadrupoles mass spectrometer (LC/MS/MS, Agilent, Santa Clara, CA). Aliquots (1 µL) were injected into the LC/MS/MS system equipped with a Symmetry C18 column (I.D. 2.1 mm, length 100 mm, particle size 3.5 µm) (Waters, Milford, MI) at a flow rate of 0.5 mL/min. The apparatus was modified based on our previous analysis (Liu et al. 2021, Wu et al. 2022). The mobile phase initially consisted of 80% solvent A (5 mM ammonia acetate in 10% methanol) and 20% solvent B (pure methanol) and was held for 0.5 min. The subsequent gradient of the mobile phase was programmed as follows: concentration of solvent B was ramped to 30% in 1.5 min, increased gradually to 95% in 6 min, then increased to 100% in 2 min, and then reduced to 20% in 1 min, and held for additional 2 min at the end. Triple quadrupoles mass spectrometer was operated in the negative-ion electrospray mode. Following the EPA Standard Method 537.1, quantitative analysis was performed by selected reaction ion monitoring (SRM) that uses two mass filters (EPA 2017). In SRM, a precursor ion is selected by the first stage of mass spectrometry (MS1), then dissociates into a molecule and a product ion, which is selected by the second stage of mass spectrometry (MS2). The precursor→product ion values were set for PFBA (213→169), PFBS (299→80), PFHxA (313→269), PFHxS (399→80), PFOA (413→368), PFOS (499→80), respectively. Calibration curves were prepared with the mixed PFAA standard to achieve a series of concentrations ranging from 0.1 to 100 µg/L using Milli-Q water. The method detection limits (MDLs) were estimated as PFBA (80 ng/L), PFBS (40 ng/L), PFHxA (80 ng/L), PFHxS (50 ng/L), PFOA (30 ng/L), PFOS (60 ng/L), respectively.

### Data analysis

Surfer 12 based on the kriging interpolation algorithm was used to generate the contour maps based on the PFAA concentrations measured in the sediments from the mesocosm study. Statistical analysis was carried out by the ANOVA test using IBM SPSS statistics 21 or the Student’s *t* test using Excel.

### Quality control and assurance

A procedural blank and a duplicate sample were analyzed in the same way as the test samples for each batch of 10 samples. Analysis of a reagent blank was also used to check the background noises. All experiments were conducted in triplicate with relative standard deviations < 15% (*n* = 3). The recoveries of the mass labeled PFHxS were in the range of 80~120%.

## Results and discussion

### Sediment-water distribution coefficients (Kd) of different PFAAs

The sediment water distribution coefficient (K_d_) was estimated in the single solute system, in which individual PFAA was spiked to an initial aqueous concentration of 100 µg/L. The *K*_d_ values of six PFAAs varied from 2.19 to 10.45, following the order: PFBA (2.19) < PFHxA (3.29) ≈ PFBS (3.48) < PFHxS (4.9) < PFOA (6.52) << PFOS (10.45). Among all six PFAAs, PFOS showed the highest adsorption potential to the sediment. As showed in Table 1, the *q*_e_ values of different compounds in the sediment showed the following sequence: PFBA (128.8 ± 4.11 ng/g) < PFHxA (168.6 ± 8.27 ng/g) ≈ PFBS (169.2 ± 4.81 ng/g) < PFHxS (190.1 ± 8.81 ng/g) < PFOA (221.5 ± 9.13 ng/g) < PFOS (247.2 ± 6.30 ng/g). In general, the PFAA adsorption to the sediment increased with the chain length and PFSAs showed greater adsorption potentials than corresponding PFCAs that are of the same chain length. These observations in this study were in good agreement with previous works on PFAA adsorption. A previous study also revealed higher sorption capacities of PFSAs onto sediments compared to PFCAs with the same carbon numbers given that PFSAs are more hydrophobic than PFCAs containing the same carbon numbers (Higgins and Luthy 2006). However, the adsorption of PFAAs was not only governed by their carbon chain lengths, but also affected by their terminal moieties. For instance, we did not observe a significant difference between the *q*_e_ values between PFHxA and PFBS (*p* > 0.05, Student’s *t* test), even though they have different chain lengths (i.e., C6 vs C4). PFAAs’ anionic moieties play a crucial role in their interaction with the sediment particles where positive functional groups, the uncharged organics, or minerals are present on the surface. Several studies showed that PFAAs may sorb onto metal oxides such as iron and aluminum oxides and associated mineral complexes including hematite, goethite, and boehmite, particularly because they pose net positive surface charge around neutral pH in the sediment (Borthakur et al. 2021). Furthermore, the low *K*_d_ and *q*_e_ values of PFBA, PFHxA, and PFBS implied the higher solubility and mobility of these short-chain PFAAs in the water (Rayne and Forest 2009).

**Table 1:** Characteristics of PFAA adsorption to the sediment in single- and dual-solute systems

		*q*_e_ in single-solute system (ng/g)	*q*′_e_ in dual-solute system (ng/g)	$\alpha$	Percent decrease (%)
PFBA-PFHxS	PFBA	128.8 ± 4.1	34.9 ± 0.8	0.271	73%
	PFHxS	190.1 ± 8.8	178.5 ± 7.9	0.939	6%
PFBS-PFHxS	PFBS	169.2 ± 4.8	49.1 ± 2.2	0.290	71%
	PFHxS	190.1 ± 8.8	148.9 ± 5.9	0.783	22%
PFHxA-PFHxS	PFHxA	168.6 ± 8.3	51.9 ± 3.5	0.308	69%
	PFHxS	190.1 ± 8.8	168.4 ± 5.3	0.886	11%
PFOA-PFHxS	PFOA	221.5 ± 9.1	90.7 ± 5.0	0.409	59%
	PFHxS	190.1 ± 8.8	77.1 ± 4.4	0.406	59%
PFOS-PFHxS	PFOS	247.2 ± 6.3	183.3 ± 6.3	0.741	26%
	PFHxS	190.1 ± 8.8	65.6 ± 5.9	0.345	65%

### Competitive adsorption between PFAAs to the sediment

**Fig. 2:**
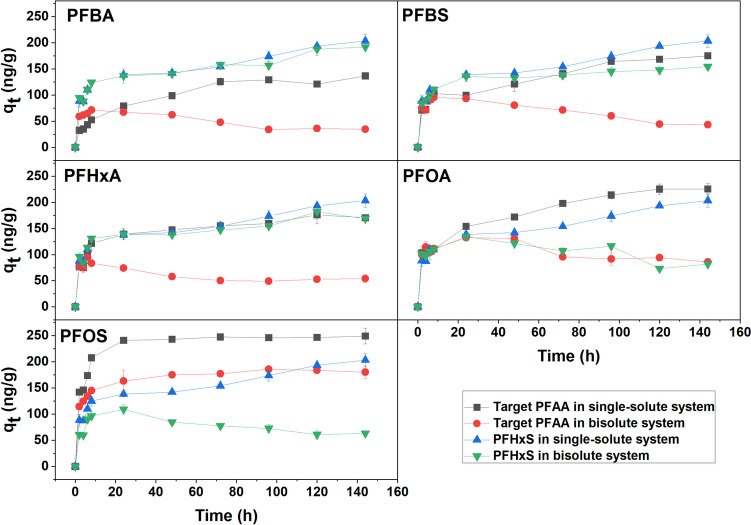
PFAA adsorption to the sediment in single and bi-solute systems. In bi-solute systems, PFHxS was dosed at an equal concentration (100 µg/L) as the target PFAA

Competitive adsorption among PFAAs was studied between PFHxS and the other five PFAAs in dual solute systems, since PFHxS exhibited a median adsorption potential to the sediment among all six tested PFAAs in the single solute batch assays. As shown in Fig. 2, the presence of PFHxS in the dual solute system negatively affected the adsorption of all other five PFAAs. On the other hand, adsorption of PFHxS also declined in the dual solute systems compared to the corresponding single solute batch. With the presence of 100 µg/L PFHxS, the q_e_ values of PFBA, PFBS, and PFHxA dropped by 69~73%. However, these three short-chain PFAAs had less negative influence on PFHxS than PFHxS did on them, as the q_e_ values of PFHxS remained 78~94% of that in the single solute system. Student’s *t* test revealed no significant difference on the impacts of PFHxS to the adsorption of PFHxA or PFBS.

In the dual solute system of PFOA-PFHxS, PFOA and PFHxS showed similar inhibition on each other with q_e_ decrease by 59%. PFOS was the most potent inhibitor to PFHxS. In the PFOS-PFHxS dual solute system, the adsorption of PFOS decreased by 26%, while PFHxS decreased by 65%, compared to those in their single solute systems. Overall, the competitive adsorption ability was ranked as PFBA < PFHxA ≈ PFBS < PFHxS ≈ PFOA < PFOS (Fig. 2). This order is in good agreement with their *K*_d_ values for the adsorption to this sediment, possibly attributed to their differences in binding affinities and interactions with the sediment surface (Lee et al. 2020).

In single solute systems, all six PFAAs exhibited two phases in adsorption: a fast adsorption within the first 8 to 24 h, followed by a slow process to reach the equilibrium for up to 6 days (Fig. 2). However, in biosolute systems, the addition of PFHxS caused the desorption of PFBA, PFBS, PFHxA, and PFOA after the initial fast adsorption phase. However, such desorption effect was not observed for PFOS in the dual solute system. For PFHxS in the dual solute systems, only PFOA and PFOS induced the desorption of PFHxS. These results validated the competition in the PFAA adsorption when they co-exist in the system. The greater adsorption potential, the stronger competition effect. This is probably due to the competition for the adsorption sites available on the surface of the mineral and organic matter in the sediment (Wen et al. 2017, Xiao et al. 2011).

### PFAA transport in a mesocosm

To mimic the transport in a static aquatic system that receives a point source of mixed PFAAs, a mesocosm was constructed with the sediment sample collected from the Hudson River. When PFAAs in the mesocosm outflow reached equilibrium after 4 weeks (28 days) of operation, mass distribution of PFAAs in the sediment were analyzed as showed in Table 2. Satisfactory total mass recoveries (90.9 to 125.6%) were achieved for all six PFAAs in the mesocosm system, indicating the mass loss was negligible through atmospheric evaporation, adsorption to the apparatus, or decay caused by biotic and abiotic reactions. Based on the mass balance in Table 2, PFAAs distributed in sediment and water in mass ratios between 1:4 and 1:2. Total mass adsorbed to the sediment for six PFAAs were ranked as: PFHxS (0.85 ± 0.02 mg, 20.4%) ≈ PFBS (0.92 ± 0.01 mg, 21.7%) < PFOA (1.02 ± 0.02 mg, 27.3%) ≈ PFHxA (1.04 ± 0.05 mg, 29.8%) < PFBA (1.12 ± 0.05 mg, 30.1%) << PFOS (1.55 ± 0.04 mg, 39.2%).

**Table 2:** Mass balance of different PFAAs in the mesocosm

	PFBA	PFBS	PFHxA	PFHxS	PFOA	PFOS
Adsorbed to the sediment (mg)	1.12 ± 0.05	0.92 ± 0.01	1.04 ± 0.05	0.85 ± 0.02	1.02 ± 0.02	1.55 ± 0.04
Dissolved in the outflow (mg)	2.81 ± 0.05	3.32 ± 0.06	2.65 ± 0.04	3.16 ± 0.05	2.37 ± 0.04	3.41 ± 0.07
Total detected in the sediment and outflow (mg)	3.93 ± 0.09	4.24 ± 0.07	3.68 ± 0.09	4.01 ± 0.07	3.39 ± 0.06	4.96 ± 0.11
Total inflow mass (mg)	3.72 ± 0.11	4.23 ± 0.12	3.49 ± 0.01	4.16 ± 0.08	3.73 ± 0.01	3.95 ± 0.13
Mass recovery (%)	105.7	100.4	105.7	96.4	90.9	125.6

PFOS showed the highest adsorption to the sediment and nearly 40% of the total discharged PFOS in the inflow partitioned into the solid phase. This aligns well with its greatest *K*_d_ value and competitive adsorption behavior as we observed. Surprisingly, PFBA was the second highest in the total adsorption amount in the sediment. This might be associated with its high hydrophilicity and small molecule size, facilitating its mobility in the sediment of the mesocosm system as evident in the detailed discussion below. Following PFOS and PFBA, PFHxA and PFOA showed similar adsorption to the sediment. Even with higher *K*_d_ and competitive ability, PFBS and PFHxS showed the lowest adsorption to the sediment, which may be restricted by their ability to diffuse into the deeper sediment.

Considering the total adsorption mass in the sediment in our mesocosm study, PFCAs were greater than their corresponding PFSAs that contain the same carbon numbers and are more hydrophobic, though with the exception of PFOS. This underlines the transport behavior of PFAAs is not merely affected by their hydrophobic interaction with the sediment, but also by the dimension of their impacted area. PFAAs that are more hydrophilic and of smaller molecule size have greater diffusion in the porous sediment than the larger molecules, leading to a deeper penetration into the sediment and the broader distribution area (Deng et al. 2010, Yu et al. 2009).

### Long-term PFAAs distribution in the mesocosm system

#### Vertical distribution

**Fig. 3:**
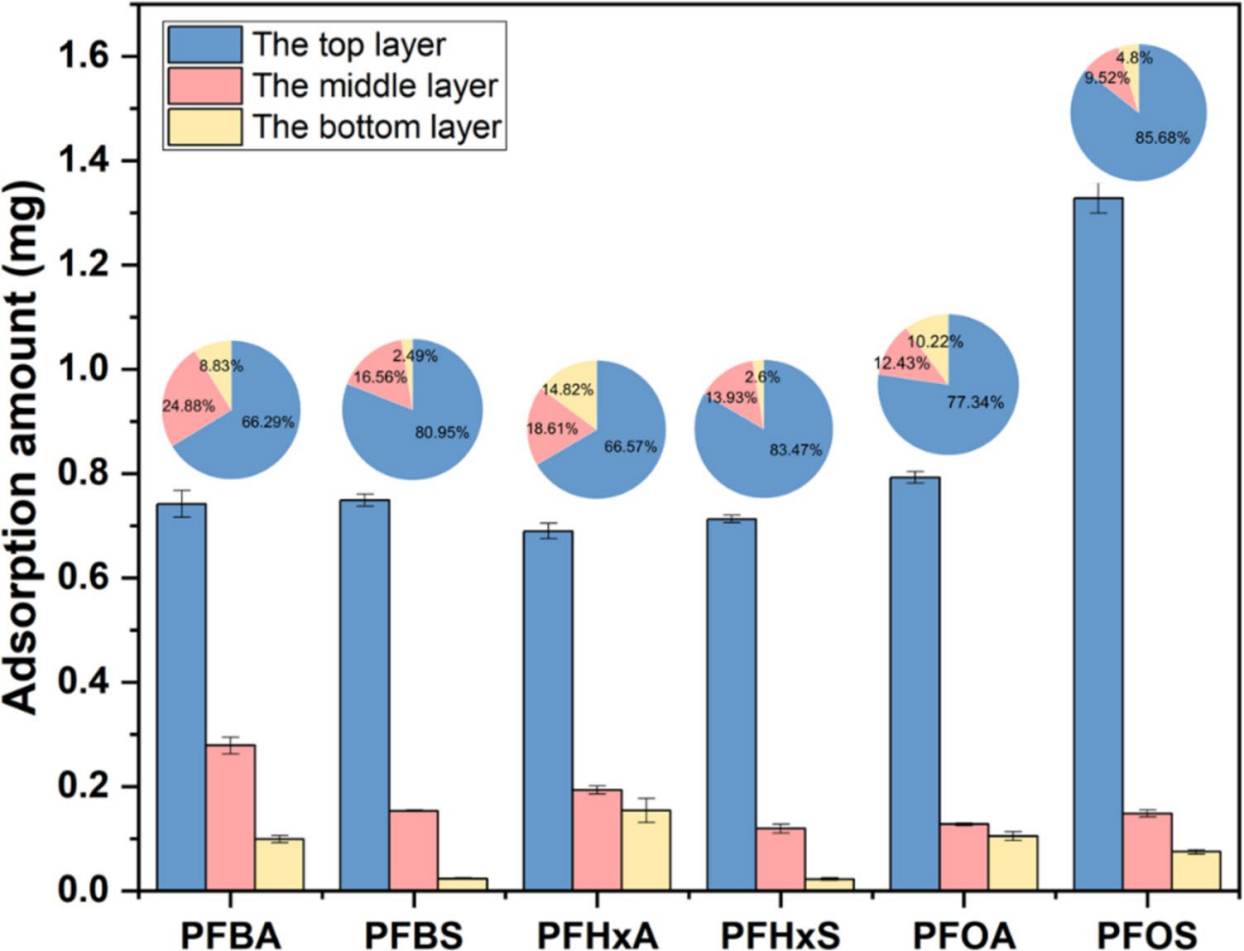
The total adsorption amount of 6 PFAAs in different layers of the sediment. Pie charts above columns indicate the proportion of PFAA distribution among three different layers

**Fig. 4:**
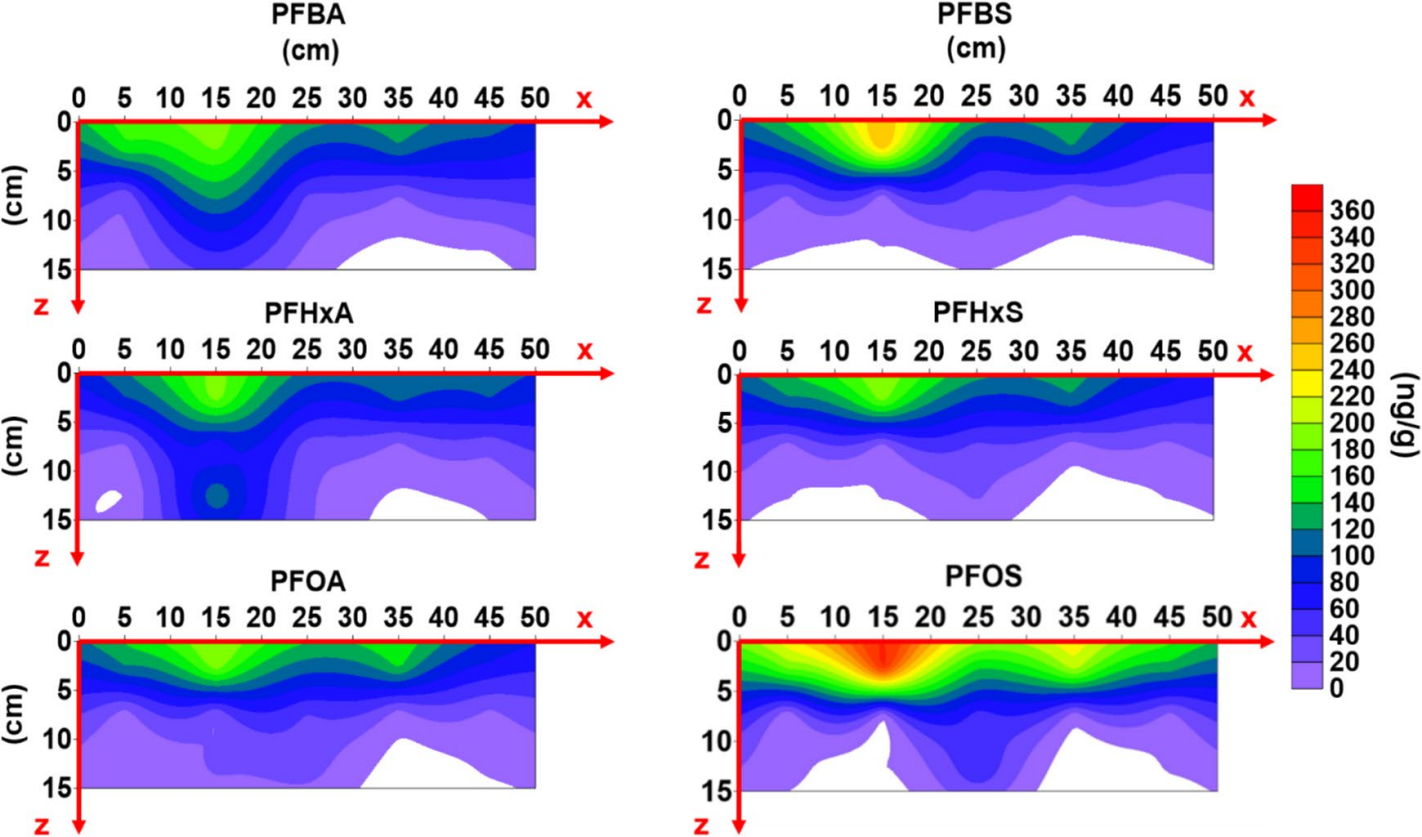
Vertical mass distribution of 6 PFAAs in the sediment (*y* = 12.5 cm). White color in the image represents PFAA concentration is below the method detection limit

To further investigate the vertical distribution of PFAAs in the sediment in the mesocosm, the total PFAA mass was summed for 3 horizontal layers: the top layer (0~5 cm), the middle layer (5~10 cm), and the bottom layer (10~15 cm) as shown in Fig. 1b. As showed in Figs. 3 and 4, the adsorption amount of the analyzed PFAAs decreased along the increase in depth. A majority of the six target PFAAs were mainly adsorbed in the top layer, which accounts for more than 65% of the total adsorbed PFAAs in the sediment. According to the ANOVA Test results, the concentrations of PFAAs in the top layer were detected at significantly higher concentrations compared to those in the middle and the bottom layer (Fig. S2).

In the top layer, PFOS showed the highest adsorption amount (1.33 ± 0.03 mg), while the other PFAAs showed similar adsorption of 0.69~0.79 mg (Fig. 3). In the middle layer, PFBA showed the highest adsorption amount (0.28 ± 0.02 mg) than the other PFAAs in the sediment. In the bottom layer, PFOS and all three PFCAs showed similar amount ranging from 0.074 ± 0.005 mg to 0.154 ± 0.023 mg, while adsorption of PFBS (0.023 ± 0.002 mg) and PFHxS (0.022 ± 0.003 mg) was minimal. Based the distribution contours in Fig. 3, it is possible that PFBA and PFHxA (and possibly PFOS) can diffuse deeper if the dimension of the mesocosm is extended in depth. Furthermore, in the bottom layer, the adsorption amount of C4 and C6 PFCAs was higher than the PFSAs with the same carbon chain length (Fig. S2). These results suggested that the functional moieties may play an important role in regulating the vertical transport of PFAAs in the sediment.

It is also interesting to note the partitioning of PFAAs in different layers. Almost 85.7% of the adsorbed PFOS was in the top layer, while only 4.8% amount in the bottom layer. Similar partitioning trends were observed for PFBS and PFHxS, even though the absolute adsorption was much lower than PFOS. PFBS, PFHxS, and PFOS in the top layer showed 31.5, 31.1, and 16.9 times higher than those in the bottom layer, respectively. In contrast, three PFCAs showed profound partitioning even in the bottom layer, and 8.8~14.8% of the total adsorbed mass was distributed in this layer (Fig. 3). Compared to the top layer, amount of adsorbed PFCAs only reduced by 3.5~6.5 times in the bottom layer. To sum up, PFSAs tend to stay in the top layers, while PFCAs can penetrate into the deeper layers without significant retardation effects.

#### Horizontal distribution

**Fig. 5:**
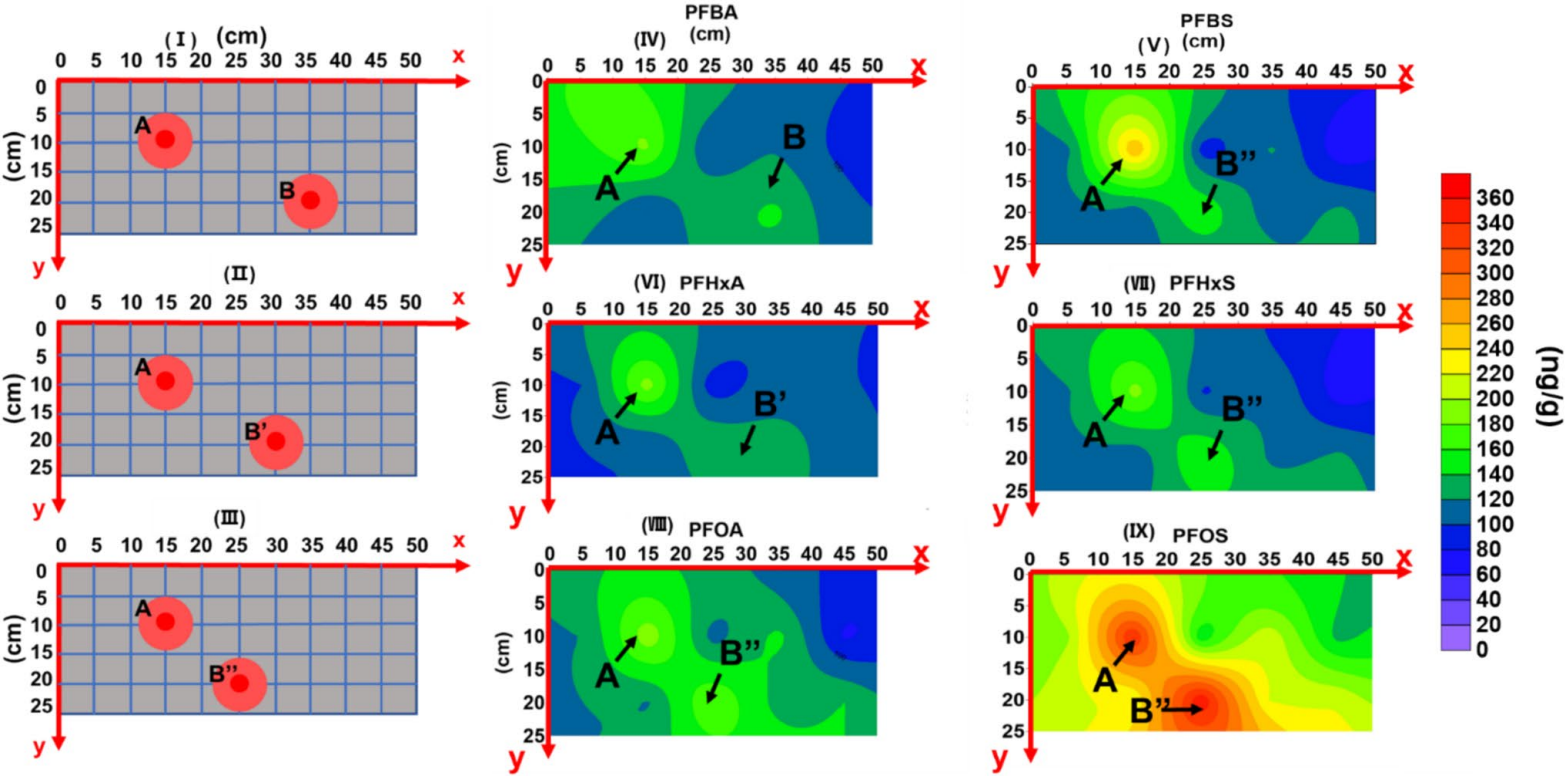
Location of two hotspots (**I**–**III**) and horizontal mass distribution (**IV**–**IX**) of 6 PFAAs on the top layer. For the first hotspot A, all 6 PFAAs co-locate at *x* = 15 cm and *y* = 10 cm. The second hotspots, B, B’, and B’’ for **I** PFBA, **II** PFHxA, **III** PFBS, PFHxS, PFOA, and PFOS, are located at *x* = 35 cm, 30 cm, and 25 cm, respectively, with the same *y* = 20 cm

To evaluate the distribution of different PFAAs in the horizontal direction, the top layer was selected as it concentrated the majority (66.29~85.68%) of the total adsorbed PFAAs. Importantly, two hotspots were found for each PFAA to concentrate in the top layer of the sediment (Fig. 5). The distance from the point pollution source to the first hotspot A was ~18 cm for all six PFAAs and its coordination is *x* = 15 cm and *y* = 10 cm. For the second hotspot B, all three PFSAs and PFOA were located ~32 cm away from the point source with the coordination of *x* = 25 cm and *y* = 20 cm (Fig. 5 III). It is interesting that this second hotspot migrated further along the x direction to 30 and 35 cm for PFHxA and PFBA, respectively (Fig. 5I and II). Thus, their distances from the point source of the system were ~36 cm for PFHxA and ~40 cm for PFBA, indicating greater mobility than the other four PFAAs. The continuous input of PFAAs into the system adsorbed in the sediment contributed to the formation of these two hotspots. It is likely that hotspot A emerged first due to its proximity to the point source and then culminated on the surface of the mesocosm until saturation. As discussed above in the “Competitive adsorption between PFAAs to the sediment” section, the competitive adsorption of different PFAAs compounds exist in the system might result in the desorption of less competitive compounds (e.g., PFBA and PFHxA) from the sediment (Fig. 2), which could migrate along the flow direction and adsorb in the sediment again at a further location, resulting in the formation of the second hotspot B.

PFAA adsorption mass at the hotspots were estimated by integration for the sediment in the radius of 5 cm from each hotspot in the top layer. The total mass of PFAAs adsorbed in hotspot A following the order of PFBA (0.057 mg) ≈ PFHxA (0.058 mg) < PFHxS (0.064 mg) ≈ PFOA (0.066 mg) < PFBS (0.079 mg) < PFOS (0.108 mg) (Table S2). In hotspot B, the adsorbed amount of different PFAAs followed the sequence of PFBA (0.048 mg) ≈ PFHxA (0.048 mg) ≈ PFBS (0.051 mg) ≈ PFHxS (0.055 mg) < PFOA (0.065 mg) < PFOS (0.12 mg) (Table S2). PFOS and PFOA showed higher or comparable adsorption amount in the first spot than the second one, while the other PFAAs showed the relatively lower concentration at the second hotspot compared to the first one. The dominance of PFOS at both hotspots was probably due to its high adsorption potential (*K*_d_) and its ability to compete and reduce the adsorption of other PFAAs. This is also in good agreement with previous studies indicating adsorption of long-chain PFAAs to the soil or sediment is through hydrophobic effects and/or surface complexation with uncharged organic and mineral surfaces (Hong et al. 2020).

Either of these two hotspots takes a volume of 392 cm^3^ (= π × (5 cm)^2^ × 5 cm), only standing for ~2.1% of the total sediment (18,750 cm^3^ = 50 cm × 25 cm × 15 cm) in the mesocosm. However, two hotspots (A and B) adsorbed 9.37 to 14.77% of the total PFAAs adsorbed in the sediment of the whole mesocosm. As shown in Table S2, the amount of PFAAs adsorbed to these hotspots was up to 4 times as high as the average adsorption amount to the sediment of the equal volume in the mesocosm. These results indicated point sources of PFAA pollution like WWTPs can create hotspots in the downstream sediment, which should be the prioritized for the remediation of the contaminated sediment. With continuous inflow of PFAAs into a system like this mesocosm, more hotspots may appear with time, especially when the dimension of the system (e.g., a river) extends in length and width.

## Conclusions

Our study set up a system that received a contamination mixture of PFAAs from a continuous point source discharge into the surface water environment. The distinctive phenomenon observed in this study indicated that understanding the characteristics of target contaminants and their interaction with the matrices is important for predicting the mobility and transport of PFASs in the environment and to develop more effective remediation/management strategies. The results from our mesocosm study demonstrated the majority of PFAAs adsorbed to the subsurface sediment, but the adsorption decreased rapidly with the increase of depth. This indicated subsurface sediment can serve as a secondary source for long-term release of PFAAs into surface water. However, short-chain PFAAs, particularly PFBA and PFHxA, showed strong potency to penetrate through the deeper sediment, conducive to a broader impact area and greater total adsorption mass. This calls for the attention to these short-chain PFAAs in deeper sediment during cleanup. PFBS and PFHxS showed the least overall contamination due to limited mobility and adsorption efficiency. The co-existence of commingled PFAAs can also profoundly affect the amount and distribution of these two PFAAs. In addition, the formation of two hotspots that concentrated PFAAs downstream from the point source provides guidance for the remediation of contaminated sediment. Once identified, these hotspots can be removed or treated with high priority to reduce the liability and cross-contamination during engineering implementations.

Note that our mesocosm design was streamlined without the flow of the main water body. Without such water flow, our system is considered lentic, in which PFAA adsorption can reach equilibrium faster and the partitioning to sediment can be condensed in a smaller dimension. When considering for lotic systems with running water (e.g., rivers and streams), the partitioning to sediment can be proportionally elongated in the horizontal direction and extended/diluted in the vertical direction. Further studies are needed using systems with water flow to gain a comprehensive view on the transport of PFAAs in lotic systems. It is also important to consider factors, such as water chemistry (e.g., pH, salinity, and DOC) and environmental conditions (e.g., temperature and flow rate) (Garg et al. 2021). The discharge of PFAAs from WWTPs and other point sources can be changing over time. Some recent studies revealed PFASs content in the aquatic environments in central and south Florida were mainly in the range of 10~300 μg/L (Li et al. 2022a, b, Yong et al. 2021). In this present study, we set 100 μg/L as the average inflow PFAA concentration. Thus, future studies should be conducted to assess the influence of inflow concentration and dynamics to PFAA distribution in the sediment.

Furthermore, as the system was set lentic without a water flow, we neglected the impacts of suspended particles for PFAA adsorption. Suspended particles can be rich in organics and significant linear relationships were previously found between PFAS adsorption and total organic carbon in suspended particles (Gao et al. 2015). The other limitation is that the PFAA distribution in the porous water was not distinguished from sediment adsorption as the collection of porous water in each sediment cube was empirically challenged. Interventions of benthic organisms can also cause major shifting in the transport of contaminants in the sediment (Li et al. 2022a). Restrictive to the test conditions and time constraints, more hotspots may occur if the mesocosm system is enlarged and/or being operated in a longer time. The competitive adsorption of PFASs with other traditional pollutants in aquatic environments also underscores future investigation.

## Supplementary Information

Below is the link to the electronic supplementary material.

Supplementary file1 (PDF 636 KB)

## Data Availability

Data will be made available upon reasonable request.
