# Supplementary material for: Distinctive adsorption and transport behaviors of short-chain versus long-chain perfluoroalkyl acids in a river sediment
Source: Environ Sci Pollut Res Int. 2024 Dec 7;31(59):66854–65. doi: 10.1007/s11356-024-35725-1 (PMC11666611; doi:10.1007/s11356-024-35725-1)
Supplement: Supplementary file 1 — Supplementary file1 (PDF 636 KB) [file 11356_2024_35725_MOESM1_ESM.pdf]

Supplementary Information

for

Distinctive Adsorption and Transport Behaviors of Short-chain versus Long-chain Perfluoroalkyl Acids in a River Sediment

Na Liu[a,b] and Mengyan Li[a]*

[a] Department of Chemistry and Environmental Science, New Jersey Institute of Technology, Newark, NJ 07102 United States

[b] National Engineering Research Center for Efficient Utilization of Soil and Fertilizer Resources, College of Resources and Environment, Shandong Agricultural University, Tai'An 271018, PR China

* Corresponding author

Phone: +1-973-642-7095

E-mail: mengyan.li@njit.edu

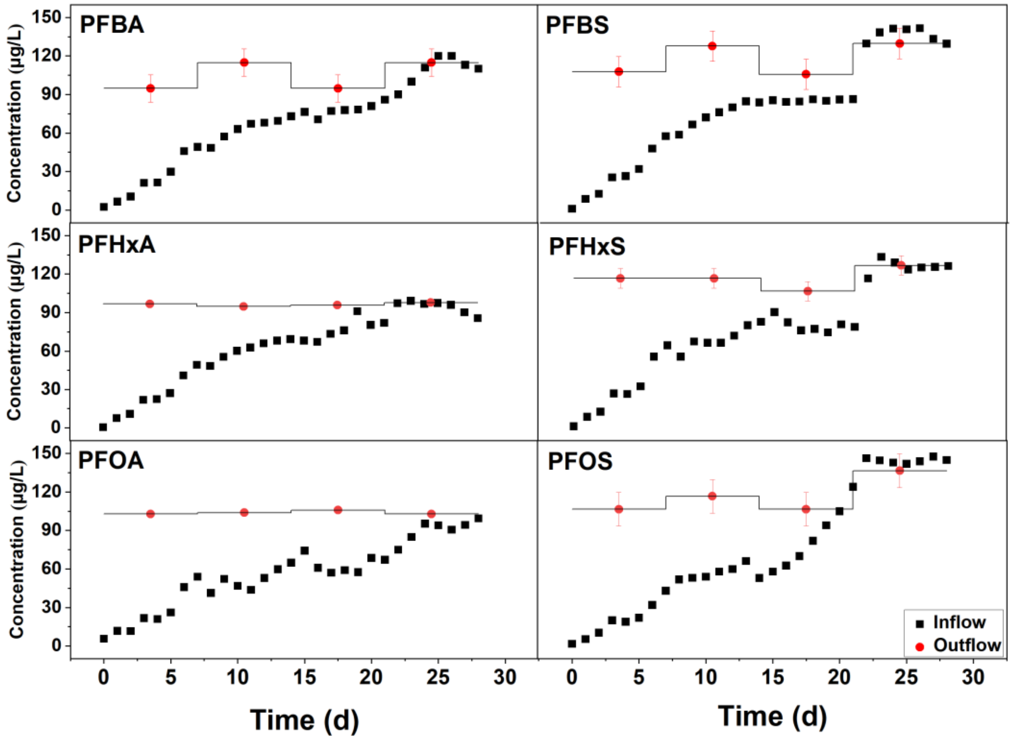

Fig. S1 PFAA concentration in the inflow (red lines) and outflow (black dots) of the mesocosm for 4 weeks.

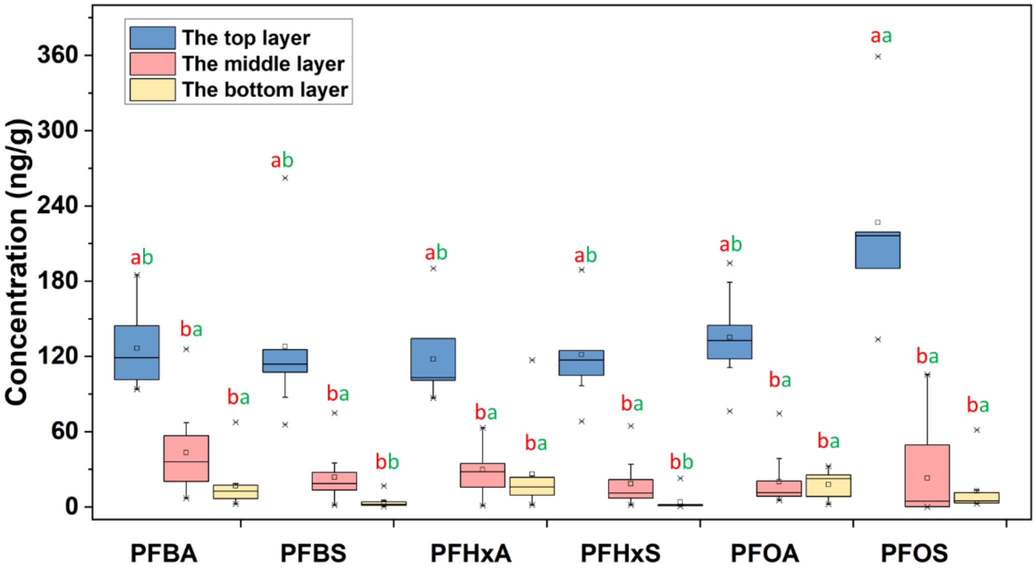

Fig. S2 Distribution of 6 PFAAs in different layers of the sediment in the mesocosm. Significant difference in PFAA concentrations between different layers and among different PFAAs in the same layer were tested using the ANOVA test ($p < 0.05$). Blue letters show significant differences between different layers. Green letters indicate significant differences between different PFAAs in the same layers.

Table S1. List of six long-chain and short-chain PFAAs and their physicochemical properties.

PFAA name	Abbr.	Chemical formula	M.W. (g/mol)	Water solubility[a] (g/L)	pKa[b]	$logk_{ow}$[b]	Molecule diameter[d] (nm)	Critical micelle concentration (mg/L)[e]	Background value in the sediment (ng/g)
perfluorobutane acid	PFBA	$CF_3(CF_2)_2COOH$	214	NA	0.4	2.32	0.22-0.60	0.76	0.55±0.04
perfluorobutane sulfonate	PFBS	$CF_3(CF_2)_3SO_3H$	300	46.2	0.14	2.73	0.28-0.87		1.04±0.01
perfluorohexane acid	PFHxA	$CF_3(CF_2)_4COOH$	314	15.7	-0.16	3.12	0.28-0.85	0.09	0.72±0.17
perfluorohexane sulfonate	PFHxS	$CF_3(CF_2)_5SO_3H$	400	1.4	0.14	4.34	0.35-1.02		1.19±0.01
perfluorooctanoate acid	PFOA	$CF_3(CF_2)_6COOH$	414	3.4	-0.2	4.59	0.23-1.11	0.01	2.32±0.13
perfluorooctane sulfonate	PFOS	$CF_3(CF_2)_7SO_3H$	500	0.57	-3.27	5.26	0.26-1.35		4.75±0.05

[a]. (Du et al., 2014)

[b] (Zhang et al., 2019)

[c] (Deng et al., 2012)

[d]. Calculated by GaussView 5.0 in pH 7, 25 ℃

[e] (Bhhatarai and Gramatica, 2010)

Table S2. PFAA adsorption amount of two hotspots and the average adsorption amount (AVE) of 6 PFAAs of the equal volume (392 cm^3) as the hotspot in the sediment.

	**PFBA**	**PFBS**	**PFHxA**	**PFHxS**	**PFOA**	**PFOS**
Hotspot A (mg)	0.057 ± 0.0002	0.079 ± 0.0002	0.058 ± 0.0002	0.064 ± 0.0001	0.066 ± 0.0001	0.108 ± 0.0003
Hotspot B (mg)	0.0481 ± 0.0002	0.0505 ± 0.0001	0.0478 ± 0.0001	0.0552 ± 0.0001	0.0652 ± 0.0001	0.1207 ± 0.0003
AVE (mg)	0.0234 ± 0.001	0.0194± 0.0003	0.0217 ± 0.001	0.0179 ± 0.0004	0.0214 ± 0.0005	0.0324 ± 0.0009

References

Bhhatarai, B., Gramatica, P., 2010. Prediction of aqueous solubility, vapor pressure and critical micelle concentration for aquatic partitioning of perfluorinated chemicals. Environmental science & technology, 45(19): 8120-8128.

Deng, S. et al., 2012. Sorption mechanisms of perfluorinated compounds on carbon nanotubes. Environmental Pollution, 168: 138-144.

Du, Z. et al., 2014. Adsorption behavior and mechanism of perfluorinated compounds on various adsorbents—A review. Journal of Hazardous Materials, 274: 443-454.

Zhang, D., Zhang, W., Liang, Y., 2019. Adsorption of perfluoroalkyl and polyfluoroalkyl substances (PFASs) from aqueous solution-A review. Science of The Total Environment, 694: 133606.
